# Fe-N catalyst derived from and supported on *Lycopodium clavatum* sporopollenin exine capsules for the oxygen reduction reaction

**DOI:** 10.1038/s41598-024-77780-1

**Published:** 2024-10-30

**Authors:** Waqas Malik, Jorge Pavel Victoria Tafoya, Szymon Doszczeczko, Ana Belen Jorge Sobrido, Andrew N. Boa, Roberto Volpe

**Affiliations:** 1 School of Engineering and Materials Science, Queen Mary University of London, London, E1 4NS UK; 2 Department of Chemistry, University of Hull, Hull, HU6 7RX UK

**Keywords:** Oxygen reduction reaction, Sporopollenin exine capsules, Iron, Carbon-nanotubes, Engineering, Materials science

## Abstract

A carbon-supported electrocatalyst, featuring carbon nanotubes anchored on 3D porous graphitic carbon, was developed with the aim to perform in the operating conditions of alkaline fuel cells and metal air batteries. The catalyst was developed *via* two steps: first powders of Sporopollenin exine capsules used as a bio-based carbon support were activated *via* CO_2_ gasification to obtain a high specific area and porosity, second the derived porous carbons were impregnated by an iron salt and a nitrogen source, to be carbonised in Nitrogen at high temperature. The prepared catalyst demonstrated an efficient oxygen reduction reaction activity showing a half-wave potential of ~ 0.775 V vs. Reversible hydrogen electrode, comparable with that of commercial 20 wt% Pt/C in alkaline conditions, a good stability after accelerated degradation testing, retaining ~ 86% of the initial limiting current density, and a higher diffusion limited current density (6.3 vs. 5.1 mA cm^− 2^) than the commercial counterpart. Overall, we show the suitability of Sporopollenin exine capsule as support for electrocatalysis and a promising methodology to develop sustainable catalysts for the oxygen reduction reaction.

**Supplementary Information:**

The online version contains supplementary material available at 10.1038/s41598-024-77780-1.

## Introduction

Energy conversion and storage devices are believed to be one of the most sustainable and capable systems to tackle the environmental deprivation linked to the high global energy consumption. Energy devices such as metal-air batteries and fuel cells are regarded as one of the most promising energy conversion systems due to their high current density^1,2^. Oxygen reduction reaction (ORR) is one of the rate-determining process in such electrochemical power sources^3^. Platinum based catalysts have proved to be excellent for ORR, however, the limited resources, high cost and poor methanol tolerance are leading issues that hinder their widespread commercial application^4,5^. As such, the development of durable, active and inexpensive electrocatalyst for ORR is one of the most critical research topics to replace platinum-based devices. Much attention has been dedicated to investigating non-precious metal cathode catalysts^6,7^. Superior ORR performance is obtained by ensuring swift mass/electron transport, high density active sites and strong structural stability^8,9^.

Among the several proposed options, it is generally agreed that the key elements required for the ORR using non-precious metal catalysts comprise of nitrogen, carbon and a transition metals such as Fe or Co. Transition metal-based nitrogen-doped porous carbon (M-N-C) materials are currently considered one of the most effective alternatives to platinum-based catalysts due to their superior catalytic activity and wide availability^10–12^. The active M-N-C composites comprise of metal atoms with subsequent N-coordination settled inside a carbon matrix and graphitic carbon shells that is generated by the catalysis of the encapsulated metal and/or metal carbide particle^9,13^. Various approaches have been used to develop M-N-C catalyst with high volumetric activity and appropriate porous structure. Nonetheless, the enhancing of the performance of these catalysts is currently limited by the lack of information on the precise sites that are active in ORR, owing to the complex composition and morphology of catalysts. In addition, mass transfer capability is another important factor of ORR activity that is linked to the porous structures of the catalysts^14^. Unfortunately, the processes commonly proposed for the synthesis of nitrogen-doped carbon usually involve complex methods, which can be expensive and challenging to scale up for industrial production. Furthermore, due to weak stability of the carbon support and weak interaction between support and particles, the active particles tend to dissociate easily from or aggregate on the surface of the carbon support thereby negatively affecting the performance of the catalyst^15^.

Carbon-based materials with high specific area, appropriate morphology and high degree of graphitisation have shown to have the capability to tackle the aforementioned challenging issues^16,17^. In particular, carbon nanotubes (CNTs) have shown to be the most promising feature where metal active sites are encapsulated by high degree of graphitic shielding shells^18,19^. In fuel cell cathodes CNT-based catalysts offer numerous advantages over the more common carbon-based materials, such as carbon black, owing to their excellent electrical conductivity and large accessible surface area^20^. The exposed assembly of CNT-based catalysts enables O_2_ mass-transfer and efficient water management, thereby ensuring a more efficient fuel cell performance^21^. Nonetheless, their electrochemical performance is affected by the limited specific surface area, the pore structure and the typically sub-optimal structure of the supported carbon. As such, the development of a sustainable and inexpensive composite material which combines the advantages of CNT and a carbon support with high porosity, surface area and high degree of graphitisation is highly desirable.

In recent years, carbon materials derived from biomass have shown great potential as catalyst or catalyst support in applications for electrocatalyst and supercapacitors^22,23^. One of the main advantages of utilising biomass is that it is readily accessible, sustainable, and inexpensive^24^. High-temperature (> 700 °C) pyrolysis of biomass typically induces a high degree of graphitisation including formation of nano-crystalline graphitic domains^25,26^. Biomass generally features a naturally hierarchical pore structure generated by its growth in nature^27^. Importantly, the presence of heteroatoms in biomass means that the impregnated/loaded species can be doped in the matrix of the biomass carbon post the annealing process^28^. This can increase the adsorption of oxygen intermediates onto the surface which enhances conductivity and the electrocatalytic activity of the catalyst^29^. However, the manufacture of heteroatom-doped carbon materials that feature good catalytic activity is challenging. The synthesis of nitrogen-doped carbon usually involves complex expensive methods, that are difficult to scale up. Furthermore, it is difficult to avoid aggregation of nanomaterials, which leads to a reduced ORR activity.

Herein, we report a sustainable and inexpensive method to synthesise a simple, effective, and green catalyst with regular morphology, suitable for ORR reaction in alkaline fuel cells. An M-N-C electrocatalyst which combined the features of a porous graphitic carbon support with CNTs was prepared in two simple steps. In the first step the biomass was activated *via* CO_2_ gasification to increase the surface area and porosity. The derived porous carbon was then impregnated with an iron salt and a source of nitrogen, before it was carbonised at high temperature. Iron (Fe) was chosen as a non-precious metal as it is inexpensive and earth abundant. Additionally, Fe can catalyse the growth of CNTs and is ORR active in carbide forms. Sporopollenin exine capsules (SpEC) were chosen as the raw biomass. SpECs have been described as one of the toughest organic compounds of natural biological origin^30^. SpECs are comprised of a biopolymer which can be extracted and utilised for various application owing to its resilient nature. Valuable properties of SpEC such as the uniformity in size distribution, a porous morphology consisting of interconnected macropores, mesopores and micropores which make up for a high specific area that can potentially increase the loading of active sites as well as reducing mass transfer limitations through the network. SpEC have also known to be highly thermally and chemically stable even in strong alkaline or acidic environments^31,32^. This stability of SpEC offers a significant advantage over other common biomass-based support materials such as chitosan, cellulose or alginate, which are affected by a significant loss in thermal and mechanical stability following metal ion loading and chemical preparation which causes of the cleavage of hydrogen bonds between the polymer strands^31,33^. In a previous report^34^ we have demonstrated the potential of a SpECs as catalyst support, able to enhance stability and the dispersion capacity of the catalyst. In the present work, a novel, synthesised M-N-C catalyst (Fe-N-aSpEC) exhibited excellent ORR electrocatalytic activity, comparable to that of platinum-based catalysts.

## Experimental

### Materials

Raw spores from *L. clavatum* were purchased from Tibrewala International Ltd (Nepal). *L. clavatum* is a highly widespread spore-bearing plant, as such this work does not involve research on endangered species. SpECs were obtained from the raw spores(100 g) by extraction via hydrochloric acid. In brief, the extraction was conducted by heating with aqueous hydrochloric acid (6 M) (500 cm^3^) at 90 °C for 2 h, as previously reported^35^. The resulting SpECs are monodispersed, with uniform size of approximately 27 μm diameter. Iron (II) acetate [Fe(O_2_CCH_3_)_2_ powder (99.9% purity), melamine powder C_3_N_3_(NH_2_)_3_ (99% purity) and methanol were all sourced from Sigma-Aldrich. For oxygen reduction reaction assessment, a commercial 20% w.w. Pt/C standard was used (Fisherbrand). Additionally, a 5% w.w. Nafion™ dispersion was used for ink preparation (Sigma-Aldrich). Finally, a pH 13 electrolyte was prepared using KOH flakes (> 90% purity, Fisherbrand).

### Synthesis method

#### Activation of SpECs

Before SpECs could be used as a catalyst support, they had to be activated to increase their stability and surface area. The activation of SpEC consisted of three steps: pre-treatment, carbonisation, and activation. This process does not require the use of corrosive or toxic chemicals, making it simple and sustainable. In the pre-treatment step, the 3 g of SpECs were heated in a ventilated oven to 300 °C in air for 6 h to fix their morphology upon reaction with oxygen. This step is key to prevent any structural damage in the following higher-temperature treatments. The obtained SpECs were then pyrolysed to 900 °C at a heating rate of 10 °C min^− 1^ in a tubular furnace under a 1 L min^− 1^ N_2_ flow. As soon as the temperature reached 900 °C, the gas flow was switched from N_2_ to CO_2_ for 3 h, with a flow rate of 0.25 L min^− 1^, for the activation. Finally, the obtained activated SpECs (aSpEC) were cooled down to room temperature in N_2_ atmosphere at a flow rate of 1 L min^− 1^.

#### Synthesis of the catalyst and control samples

: For the synthesis Fe-N-aSPEC, 0.15 g of melamine and 0.056 g of iron(II) acetate were mixed and dispersed in 40mL of methanol. 0.5 g of aSpEC was then added to the solution. The resulting mixture was stirred for 24 h at room temperature, followed by drying at 60 °C in air until the methanol was completely evaporated. Once dried, the sample was pyrolysed at 800 °C, at a 10 °C min^− 1^ heating rate for 3 h in a tubular furnace under N_2_ (flow rate of 1 L min^− 1^) and then cooled to room temperature. Three control samples of the catalyst Fe-aSpEC, N-aSpEC and F-N were also synthesised via the same method but without including melamine, iron acetate and aSpEC respectively.

### Physicochemical characterisation

X-ray diffractometry (XRD) was used to investigate crystallinity and structural information of the samples using a Siemens D5000^®^powder diffractometer with aq/2q geometry in reflection mode equipped with a Cu Kα source with a wavelength of 1.540 *Å*. Characterisation of surface morphology was conducted by field emission scanning electron microscopy (SEM, FEI InspectF50^®^). HRTEM images were recorded by Jeol JEM-F200 Cold-FEG S/TEM microscope, operated at 200 kV. Energy dispersive spectra (EDS) were collected using JEOL JED Dual EDS Detectors. X-ray photoelectron spectroscopy (XPS, Thermo Scientific, Nexsa^®^) was used to investigate the chemical composition of the samples. Raman spectroscopy (Renishaw in Via^®^, 442 nm) was employed to determine defect/graphitic ratio (I_D_/I_G_). Finally, N_2_ sorption isotherms were obtained at 77 K using an adsorption Nova Quantachrome^®^ instrument. The surface area measurements were calculated by the Brunauer-Emmet-Teller (BET) method. Pore size distributions were determined by the slit pore, Quenched Solid Density Functional Theory (QSDFT) equilibrium model.

### Electrochemical measurements

Electrocatalytic activity towards dioxygen reduction in alkaline media was assessed by performing cyclic voltammetry (CV), linear sweep voltammetry (LSV) and electrochemical impedance spectroscopy (EIS). For these measurements, a glassy carbon rotating disk (RDE, 3 mm diameter), and rotating ring disk (RRDE, 5 mm diameter) working electrodes were modified by drop-casting the vitreous carbonaceous surface with an ink of 2 mg mL^− 1^ concentration of electrocatalytic material. For completing the half-cell setup, a Hg/HgO electrode (0.098 V vs. RHE, Metrohm) was used as reference electrode, and a glassy carbon rod (10 cm, Metrohm) as counter electrode. The three mentioned electrodes were connected to an Autolab PGSTAT302N potentiostat and immersed into a flask containing 50 mL of 0.1 M KOH solution saturated with high purity O_2_ for ORR assessment, and oxygen depleted using Ar for baseline correction of the electrochemical measurements. For comparison, a 20% Pt/C was used as benchmark material for ORR activity. The 2 mg of electroactive materials were weighted using an analytical balance (Fisher Scientific, 0.1 mg resolution), and mixed with 700 µL of deionised water, 264 µL of absolute ethanol and 36 µL of a 5% w.w. Nafion dispersion (Sigma-Aldrich). The obtained dispersions were sonicated with an ultrasonic microprobe applying sonic pulses of 140 W for 15 min to obtain a homogenously dispersed suspension. As mentioned previously, the glassy carbon surface of RDE and RRDE were modified by carefully depositing 5 µL and 15 µL of the sonicated inks respectively, and subsequentially evaporating the ink solvents by rotating the electrodes at 800 RPM for at least 45 min. The final mass loading of electrocatalyst on the GC electrode was fixed to ~ 0.141 mg cm^− 2^. Cyclic voltammograms were measured without rotation of the RDE and RRDE electrodes using an electrochemical potential window between 0 and 1.0 V vs. RHE. Linear sweep voltammograms were measured the same potential window at rotation speeds of 800, 1000, 1200, 1400, 1600, 2000 and 2500 RPM to enhance dioxygen mass transport towards the working electrode surface. For the LSV measurements using RRDE, a fixed potential of 1.1 V vs. RHE was applied to measure the oxidative current of undesirable hydrogen peroxide and determine effective electron transfer number and peroxide formation. Finally, electrochemical impedance spectroscopy was measured at a fixed potential of 0.5 V vs. RHE, mass transfer-controlled plateau region of the obtained LSVs, using a perturbation amplitude of 3 mV_rms_ and of frequency between 100 kHz and 100 mHz, recording 10 points per decade of frequencies. All the electrochemical potentials were adjusted using ohmic drop correction corresponding to a maximum of 5% corrected potentials^34^. To normalize the obtained current responses were converted to current density by calculating the coefficient of current expressed in mA and the geometric area of the different glassy carbon electrodes. This procedure is previously described in one of the authors articles^34^. Furthermore, Tafel slope analysis was performed by plotting Log j vs. η plot, and subsequently perform a linear fitting on the mixed controlled region (kinetic and mass-controlled region) between onset (determined for each sample when the current density reaches a value of 0.1 mA cm^− 2^) and half-wave potentials, shown in Figure S6. Each fitting was set to at least reach a value of r^2^ ≥ 0.95 to ensure linearity of plotted data^34^.

Additionally, the stability and durability of 20% Pt/C and our best electrocatalyst were assessed following the accelerated degradation testing according to the recommendation of the US Department of Energy^36^. An initial CV and LSV measurements were recorded in oxygen saturated alkaline electrolyte. Then, the working electrode was stressed by measuring without interruption 3000 CVs using a potential window between 0.6 and 1.2 V vs. RHE, measuring a final LSV for comparison with the initial electrochemical behaviour.

Finally, the presented results are the average of 3 measurements taken under same conditions. Standard deviation was calculated and multiplied by a cover factor of k = 2, to ensure at least a 95.45% of certainty. Each point of the electrochemical was analysed and set to be located between ± 2σ to corroborate reproducibility.

## Results and discussion

The synthesis of the aSpEC-based M-N-C catalyst involved a one-pot method. The metal precursor [iron (II) acetate] was mixed with melamine and via solution in methanol adsorbed onto the SpECs, followed drying by carbonisation at 800 °C for 3 h. During high temperature pyrolysis, the uniformly distributed iron particles transform mainly into metallic iron or react with the available carbon from the biomass (aSpEC) to produce carbide species.

These metallic and carbide phases contribute to the catalytic graphitisation of carbon via etching^37^. Melamine was added to the mixture to increase the nitrogen doping and thus enhance the catalytic performance by increasing the number of active sites^38^. The presence of nitrogen aids the developing of a combination of nitrogen and carbon atoms, as the melamine gets absorbed and onto the surface of the carbon encapsulated iron particles^39^. The nitrogen-carbon atoms get dissolved and diffused in liquid metastable carbides, which leads to the formation of nitrogen-doped CNT generated by the precipitation of carbon generated from the thermally decomposing particle. This synthesis strategy is referred to as gas-liquid-solid (VLS)^40^, which we used to generate a composite structure of Fe-N-CNT supported on aSpEC. For comparison, Fe-free, N-free and SpEC-free samples were prepared following the same preparation method.

### Morphology and structural characterisation

**Fig. 1:**
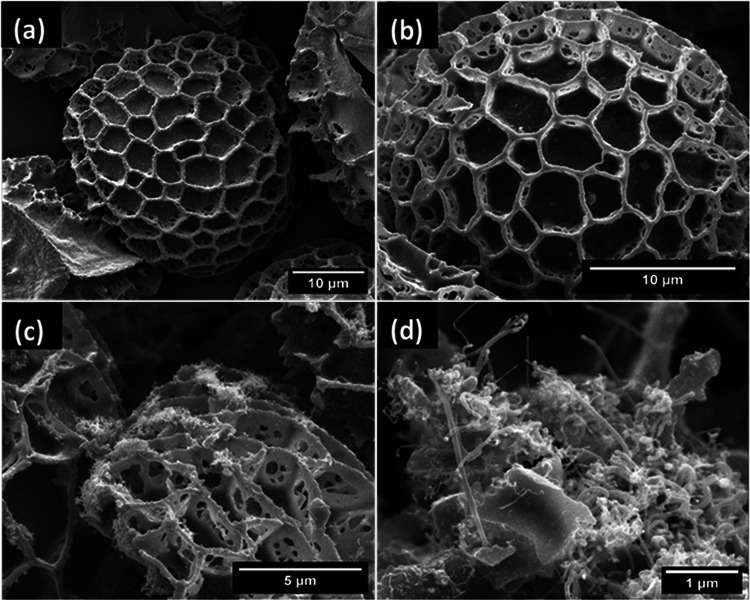
Scanning electron microscopy of (**a**) untreated SpEC, (**b**) aSpEC, (**c**) Fe-N-aSpEC and (**d**) CNT on the surface of Fe-N-aSpEC.

**Fig. 2:**
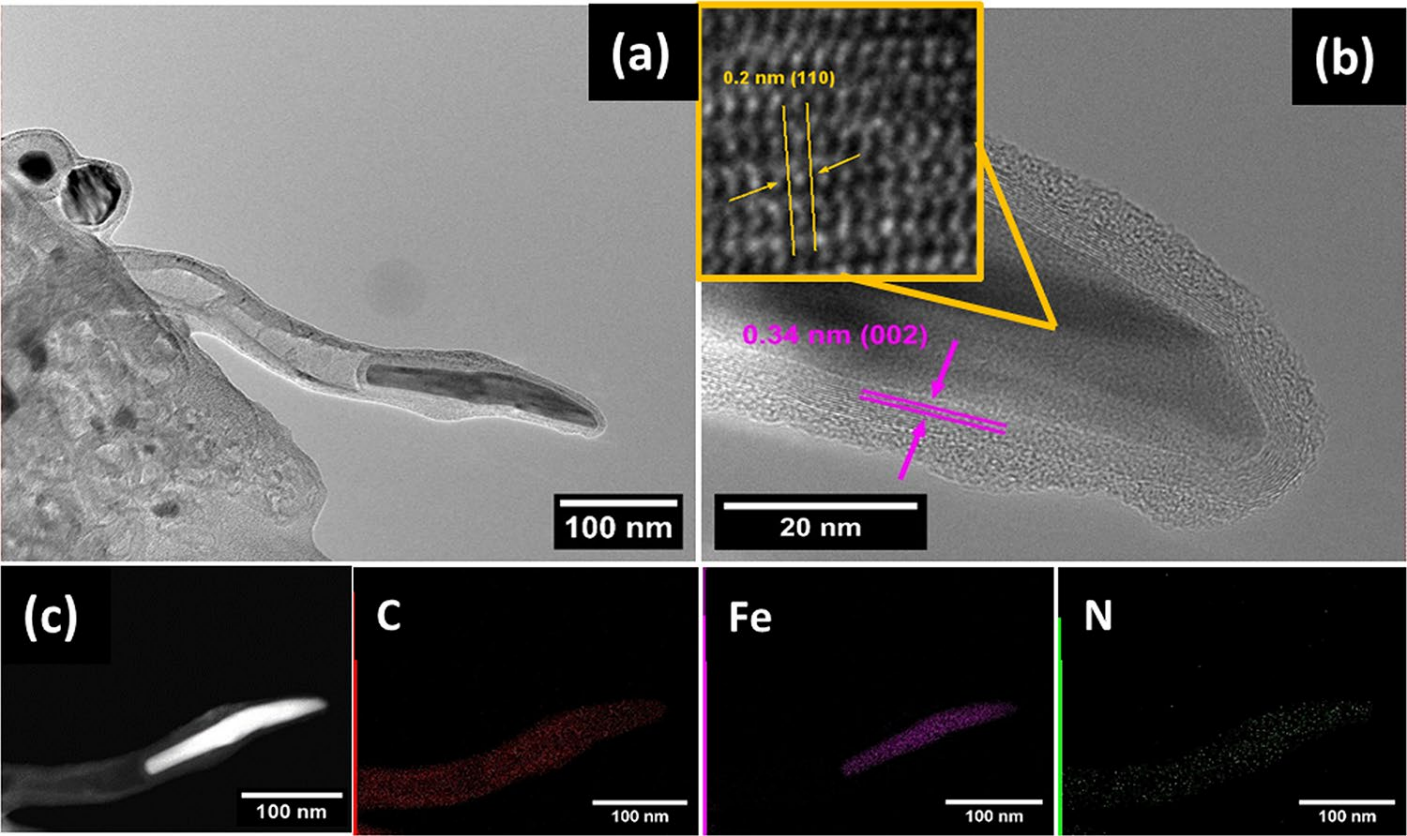
(**a**) HRTEM images of Fe-N-aSpEC. (**b**) HRTEM image of zoomed in CNT on Fe-N-aSpEC, highlighting the interlayer spacing and the planes. (**c**) HRTEM image of zoomed in CNT on Fe-N-aSpEC and its corresponding elemental mapping of C, Fe and N.

Scanning electron microscope (SEM) images revealed that the aSpEC (Fig. 1b) retained their characteristic morphology (from untreated SpEC (Fig. 1a)), consisting of interconnected uniform honeycomb-like microstructures even after the activation treatment. The Fe-N-aSpEC catalyst also retained most of the original SpEC morphology and structure (Fig. 1c). The presence of 20–30 nm diameter and 500–2 μm length CNTs, randomly distributed on the surface of the aSpEC could be detected (Fig. 1d). These CNTs offer additional active sites for electrochemical reactions and promote higher electronic conductivity, consequently enhancing both reaction kinetics and electrocatalytic activity^17^. Figure 2 displays an HRTEM images focusing on a single multi-walled CNT, with a worm-like Fe particle encapsulated in a graphene shell, which is a result of Fe-catalysed growth. The Fe particle is encapsulated at the tip of the CNT and has a diameter of around 40 nm and around 200 nm in length. Other Fe particles can also be observed which are encapsulated in graphitic shells but did not develop into CNT’s (Fig. 2a).

HRTEM images in Fig. 2b shows the iron nanoparticle with lattice fringes of about 0.20 nm, which can be assigned to the (110) plane of metallic Fe^41^. Additionally, these iron particles were encapsulated by highly crystalline graphene multi-layered shells consisting of around 10–20 layers. The interlayer spacing of the graphene layers are around 0.34 nm, corresponding to the (002) graphite planes, which is in accordance with the experimental results of free-standing few-layer graphene films^42^. Figure 2c displays HRTEM images, and the corresponding EDX mapping images reveal the distribution of carbon (C), iron (Fe) and nitrogen (N) in the Fe-N-aSpEC catalyst. Nitrogen was homogeneously distributed all through the CNT, confirming that the nitrogen was effectively doped in to the CNTs. The image also provided evidence of the iron particle being embedded in the nitrogen doped CNT. The encapsulation of the iron particle by the graphitic layer may lead to higher durability and stability, as the graphitic layers have shown to prevent corrosion of the particle from the electrolyte and help avoid induced agglomeration^39^.

### XRD characterisation

**Fig. 3:**
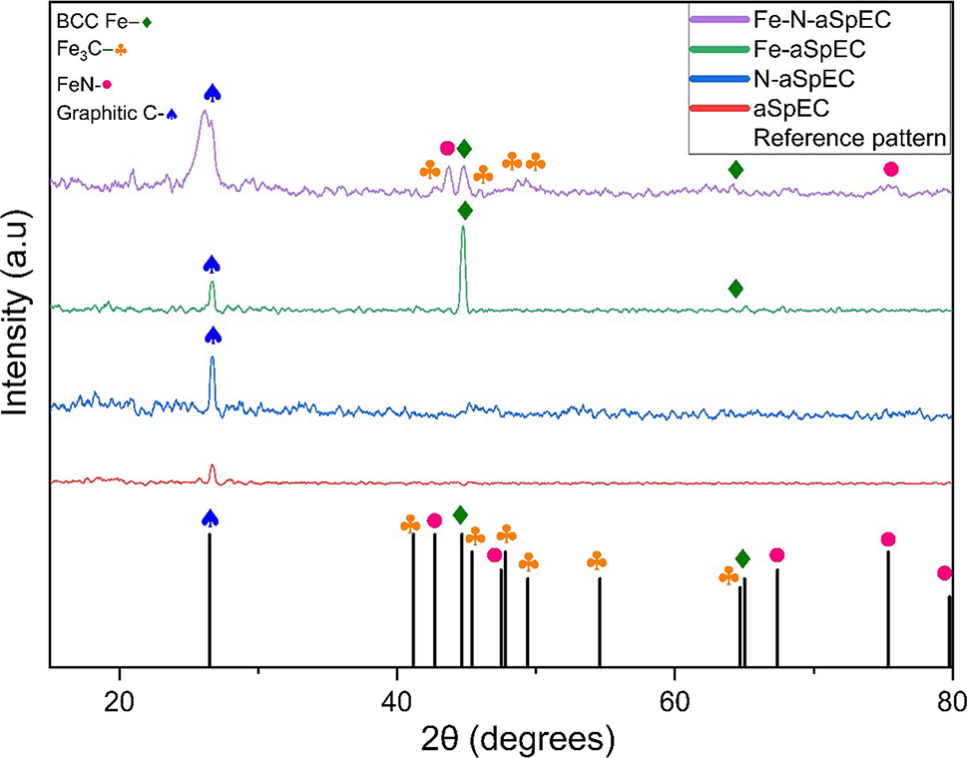
XRD patterns of Fe-N-aSpEC, Fe-aSpEC, N-aSpEC and aSpEC with ICDD standards of BBC metallic Fe, Iron carbide, Iron nitride and graphitic carbon presented below.

XRD spectra of Fe-N-aSpEC and control composites prepared are displayed in Fig. 3. The prominent peak around 26° is present in all samples and is attributed to carbon (ICDD number 041-1487) indicating a high degree of graphitisation. Fe-N-aSpEC produced a more pronounced graphite peak compared to aSpEC, Fe-aSpEC and N-aSpEC, implying a greater extent of graphitisation and potentially superior conductivity.

Both the Fe-N-aSpEC and the Fe-aSpEC produced diffraction peaks situated at 44.8° that is attributed to metallic Fe (ICDD number 006-0696). The intensity of the metallic Fe peak is higher in the Fe-aSpEC sample compared to the Fe-N-SpEC as it has a higher proportion of iron. Fe-N-SpEC also displays some Fe_3_C peaks (ICDD number 04-003-6492) located at 42.7°, 45.9°, 48.5° and 49.2°. The Fe and Fe_3_C species were developed as the available carbon from the aSpEC support reacted with iron precursor and reducing gasses (H_2_ and CO) produced via the thermal process^43^. Additionally, the diffraction peaks at 43.7° and 74.8° in the XRD of Fe-N-SpEC sample can be referenced to FeN_0.0324_ (ICDD number 075-2127). These peaks suggest that the iron is well dispersed in the Fe-N-aSpEC sample and has bonded with other available atoms to form Fe_x_-C species Fe-N_x_, while the remaining iron has remained in its metallic form.

### Raman characterisation

**Fig. 4:**
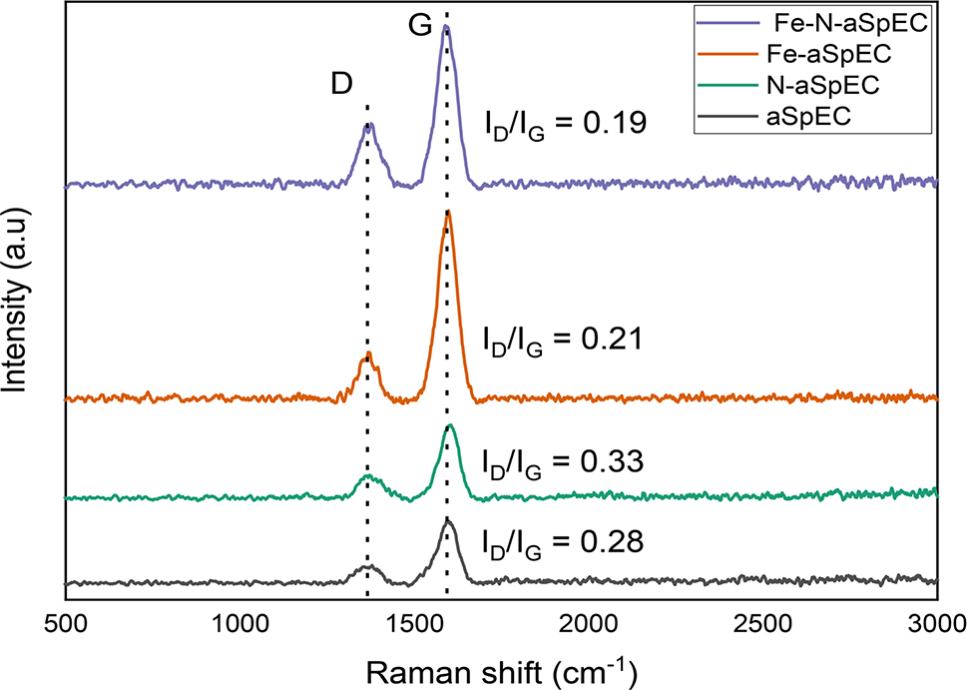
Raman spectra and I_D_/I_G_ ratios of Fe-N-aSpEC, Fe-aSpEC, N-aSpEC and aSpEC.

Raman spectroscopy (Fig. 4) was conducted to evaluate the degree of graphitisation of the prepared samples. All samples produced two distinct peaks centred at around 1360 cm^− 1^ and 1596 cm^− 1^ corresponding to the D and G band respectively. The D band correlates to the vibrations of sp3 bonds that are associated with the bonded carbon atoms in disordered graphite^44^. Whereas the G band, corresponds to the vibrations of sp^2^ hybridised carbon bonds in highly organised graphene and graphitised materials^45^. The intensity ratio of the D and G band (I_D_/I_G_ ratio) is used to quantify the defects of carbon-based material, to evaluate the level of disorder that exists in the carbon structure. Low I_D_/I_G_ ratio is associated with a higher degree of graphitisation^46^, and a decrease in amorphous sp2-bonded carbon. The I_D_/I_G_ ratios show a higher level of graphitisation in samples with Fe (Fe-N-aSpEC and Fe-aSpEC). This was expected as numerous studies have shown the effect of Fe catalysed graphitisation of carbon^34,47^. Both these samples had low and relatively similar I_D_/I_G_ ratios that could aid in enhanced conductivity, especially of the Fe-N-aSpEC catalyst. aSpEC and N-aSpEC also displayed some degree of graphitisation, which occurred during the CO_2_ activation and the pyrolysis of the SpEC support.

### Textural properties

**Fig. 5:**
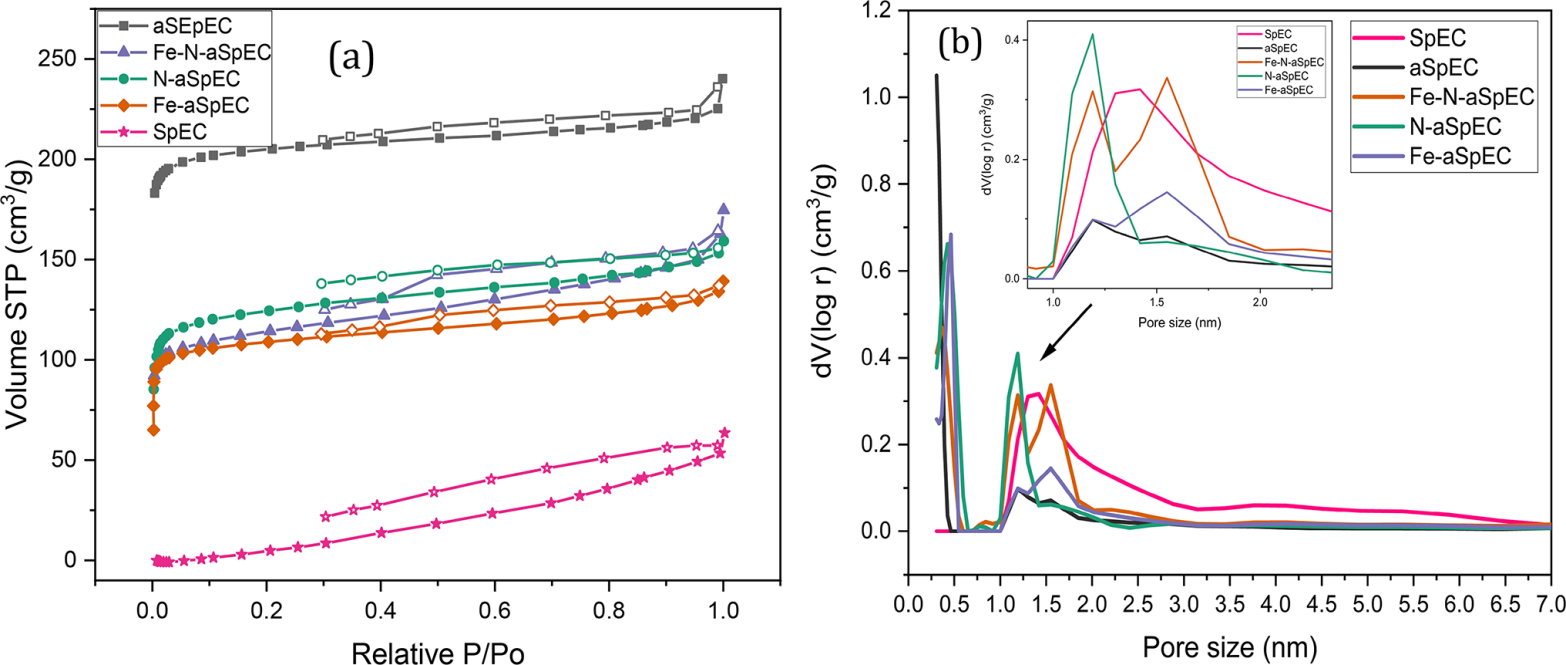
(**a**) N_2_ adsorption and desorption isotherms of all the composites. (**b**) Pore size distribution of all the composites.

Nitrogen adsorption-desorption isotherms, pore size distribution and the textural characteristic properties (surface area and pore volume) conducted by BET measurements are presented in Fig. 5a and b; Table 1 respectively. Both untreated SpEC and aSpEC samples demonstrate type IV adsorption-desorption isotherms. The hysteresis loop of untreated SpEC belongs to type H4 with noticeably elevated N_2_ filling at *P/P*_*0*_ > 0.8, indicating the presence of both slit-shaped mesopores and some micropores. On the other hand, aSpEC belongs to a hybrid H3 and H4 hysteresis loop, signifying that a mixture of slit-shaped and wedge-shaped mesopores and micropores comprise most of the porous structure of the materials.

This is in line with literature, as the H4 and H3/H4 hybrids loops are usually represented by activated carbons and other nonporous absorbents^48^. As seen in Table 1, the untreated SpEC have a low surface area and pore volume, which is unfavourable for a catalyst support. Conversely, aSpEC show increased surface area and pore volume which favour a more uniform dispersion of metal particles and prevent agglomeration. Through CO_2_ activation both the surface area and pore volume increased in aSpEC from 38 m^2^g^− 1^ and 0.084 cm^3^ g^− 1^ to 1073 m^2^g^− 1^ and 0.322 cm^3^ g^− 1^ respectively. The catalyst and the control samples also demonstrate type IV adsorption-desorption isotherms (Fig. 5a). In comparison, the three catalysts produced a hybrid of H2 and H3 hysteresis loops instead, suggesting the presence of inkbottle-shaped pores as well as the wedge-shaped pores. As expected, a marginally higher surface area was produced by the catalysts with iron (Fe-N-aSpEC and Fe-aSpEC) compared to the catalyst with no iron (N-aSpEC), as attributed to the ability of iron to etch the carbon matrix^49^. The average pore size of untreated SpEC was mainly found to be 1.5 nm (Fig. 5b). However, a significant amount of mesopores ranging 2–6 nm were also detected, as shown by the subtle plateau in the graph of untreated SpEC. aSpEC and the three synthesised catalyst predominately consisted of micropores, with two main peaks located at around 0.5 nm and another ranging at 1–2 nm. The intense uptake of N_2_ in the isotherm of these samples at low-pressure (*P/P*_*0*_ < 0.45) demonstrates the existence of micropores, thus backing up the results reported in Fig. 5a. Indeed, a high surface area and pore volume leads to efficient ion transportation and to a strong electrocatalytic activity for the ORR^50,51^. There was minimal difference in surface area and pore volume among the doped samples, though the very slightly higher surface area observed in Fe-N-aSpEC was likely due to the presence the N-doped CNTs.

**Table 1:** Surface area and pore volume of all the composite samples.

Sample	Surface area (m^2^ g^-1^)	Pore volume (cm^3^ g^-1^)
SpEC	38	0.084
aSpEC	1073	0.32
Fe-N-aSpEC	496	0.23
Fe-aSpEC	462	0.19
N-aSpEC	457	0.22

#### XPS characterisations

**Fig. 6:**
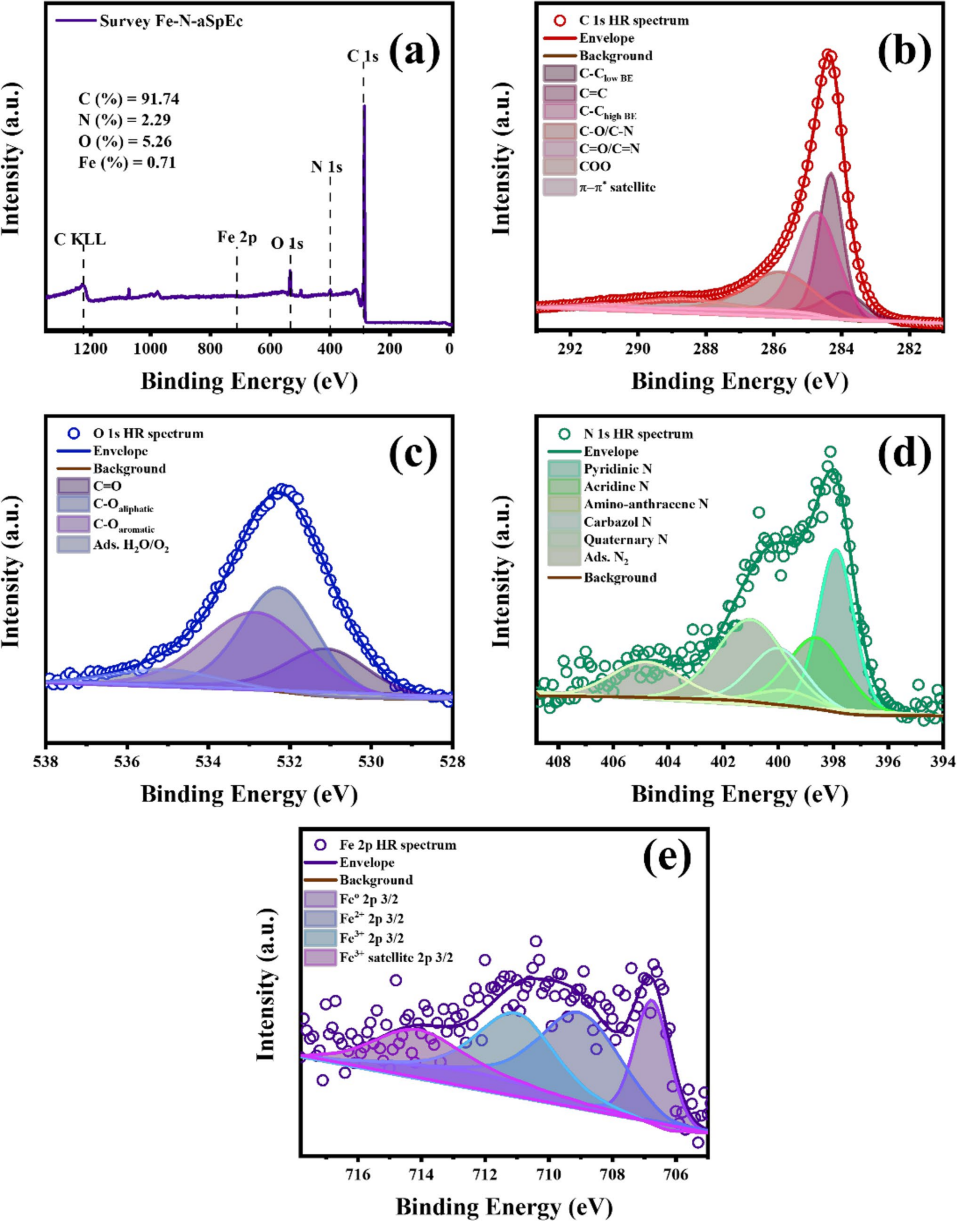
X-ray photoelectron spectroscopy characterisation of Fe-N-aSpEc sample: (**a**) survey spectrum, (**b**) C 1s spectrum, (**c**) O 1s spectrum, (**d**) N 1s spectrum, and (**e**) Fe 2p spectrum.

XPS surface analysis was used to explore the chemical environment of the SpEC, aSpEC, Fe-N-aSpEC, N-aSpEC, and Fe-aSpEC samples. In Fig. 6a, and from Figure S1a to Figure S4a in Supporting information, the survey spectra of the Fe-N-aSpEC, pristine SpEC, aSpEC, N-aSpEC and Fe-aSpEC are shown respectively. This analysis depicts a dominant signal from C 1s peak ~ 284 eV which is reasonable with the carbonaceous nature of the biomass derived material. In addition to the C 1s signal, three peaks related to N 1s, O 1s, and Fe 2p signals were found ~ 399, ~ 530, and ~ 710 eV respectively^52–55^.

The atomic quantification analysis is displayed in Table 2, and shows the dominance of C At.%, that correlates to the carbonaceous nature of most of the starting materials used for sample preparation, specifically, the untreated SpEC and melamine.

For further understanding of the activation, carbonisation and nanotube growing processes, C 1s peak fitting was performed in each spectrum of each sample using a 7 peak fitting protocol^52,53^. The peak fitting analysis for all the biomass-derived samples (Fig. 6b, and from Figure S1b to Figure S4b), showed a dominant C = C peak, (284.4 ± 0.1 eV, FWHM ~ 1.4 eV) correlated with sp^2^ hybridised C. Additionally, contributions corresponding to C-C low binding energy defective domains (~ 284 eV, FWHM ~ 1.4 eV) and C-C high binding energy defective domains which overlaps with adventitious C (~ 284.8 eV, FWHM ~ 1.4 eV) were also observed. For all the biomass-based catalysts, C 1s peak fitting presented a considerable contribution from the signal around the tail features of the spectra, corresponding to C-O/C-N (~ 286.2 eV, FWHM ~ 1.8 eV) overlap. Small contributions from C = O/C = N (~ 287.4 eV, FWHM ~ 1.8 eV) overlap, COO (~ 288.9 eV, FWHM ~ 2.5 eV) and π-π^*^ delocalised electrons (~ 292 ± 1 eV, FWHM ~ 3.5 eV) were also assigned, indicating a small contribution of complex O and N functionalities bonded with sp^2^ C, and the presence of graphitic domains with high concentration of electron clouds perpendicular to the graphene-alike planes facilitating electron mobility (Fig. 6c and from Figure S1c to Figure S4c)^52,53^. N 1s peak fitting was also performed on the nitrogen doped samples (Fe-N-aSpEC and N-aSpEC), considering 6 different N contributions, Fe - N_4_/pyridinic overlap, acridine N, amino-anthracene N, carbazole N, quaternary N and adsorbed (Fig. 6d and Figure S3d). The peak centre of each N signal was constrained to the general accepted values: 397.9, 398.7, 399.7, 400.3, 401 and 404 eV respectively (FWHM ~ 2 eV)^56,57^. The most prominent signal in Fe-N-aSpEC sample is the 397.9 eV, indicating the preferential formation of Fe-N/pyridinic N, expected from the design of the sample preparation. Moreover, the tail component of the N 1s spectrum shows the dominant contribution from graphitic N domains, indicating the successful formation of N doped graphitic domains on the surface of the CNTs and the graphitised surface of aSpEC derived from melamine (Fig. 6d). In comparison the N content of N-aSpEC shows a similar trend as Fe-N-aSpEC. Nonetheless for the N-aSpEc sample, the origin of the 397.9 eV signal is only attributed to pyridinic N. A noticeable decrease on the contribution of 401 eV, can be observed, which relates to the graphitic N in this sample, compared to the Fe-containing sample. These findings indicate that in presence of Fe, the degree of formation of N-doped graphitic domains is enhanced and therefore positively contributing to a decrease in onset potential (pyridinic N) and diffusion limiting current density (graphitic N) of our best performing electrocatalyst. This is also in accordance with Raman spectroscopy characterisations^58–60^.

Additionally, peak fitting was performed in the Fe 2p high resolution region for Fe-N-aSpEC, and Fe-aSpEC (Fig. 6e and Figure S4d). The peak fitting was performed using a typical 4 doublet scheme, Fe^2+^ doublet, Fe^3+^ doublet, and their correlated satellite doublets^55,61,62^. For the mentioned samples, the Fe 2p high resolution peak showed the existence of a mixture of metallic Fe, Fe^2+^ and Fe^3+^ oxidation with their characteristic satellites. The presence of Fe^0^ peak at 706.8 eV (FWHM ~ 2.8 eV) is correlated with the presence of Fe^0^ nanoparticles, as shown in TEM, which acted as catalyst for the growth of N-doped CNTs on the surface of aSpEC. The Fe^2+^ signal can be reasonably attributed to the coordination of Fe with C and N. Moreover, the Fe-aSpEC sample shows the formation of Fe nanoparticles, Fe^2+^ and Fe^3+^, implying the formation of Fe_x_-C species embedded on the aSpEC surface as shown in XRD characterisation, which was reported previously by this research group^34^.

Finally, O 1s peak fitting was performed for all the samples, considering the contribution of C = O, C-O aliphatic, C-O aromatic and adsorbed O_2_ signals, centred at 531, 532.4, 533 and 535 eV (FWHM ~ 2.4 eV) respectively^52^. It is noticeable that the O 1s signal for SpEC (Figure S1c) is dominated by C-O aliphatic and aromatic species, which is in coherence with the proposed molecular structure of this biopolymeric structure^63^. Whereas for the aSpEC sample, the oxygen content (Table 2) is decreased after the activation process due to the thermal reduction in presence of CO_2_, reducing specifically aliphatic and aromatic O species (Figure S2c). The Fe-N-aSpEC, N-aSpEC and Fe-aSpEC samples do not show a Fe-O shoulder around 529 eV, indicating that the impregnated Fe did not react during the thermal annealing to form undesirable iron oxides, and instead, Fe ion and species got embedded into the carbonaceous matrix of the biomass derived composites^64^.

**Table 2:** Atomic percentage of SpEC, aSpEC, Fe-N-aSpEC, N-aSpEC, and Fe-aSpEC.

Material	C At. %	*N* At. %	O At. %	Fe At. %
SpEC	86.82	---	13.18	---
aSpEC	94.91	---	5.09	---
Fe-N-aSpEC	91.74	2.29	5.26	0.71
N-aSpEC	91.44	3.85	4.72	---
Fe-aSpEC	93.93	---	4.87	1.21

### Electrochemical analysis

**Fig. 7:**
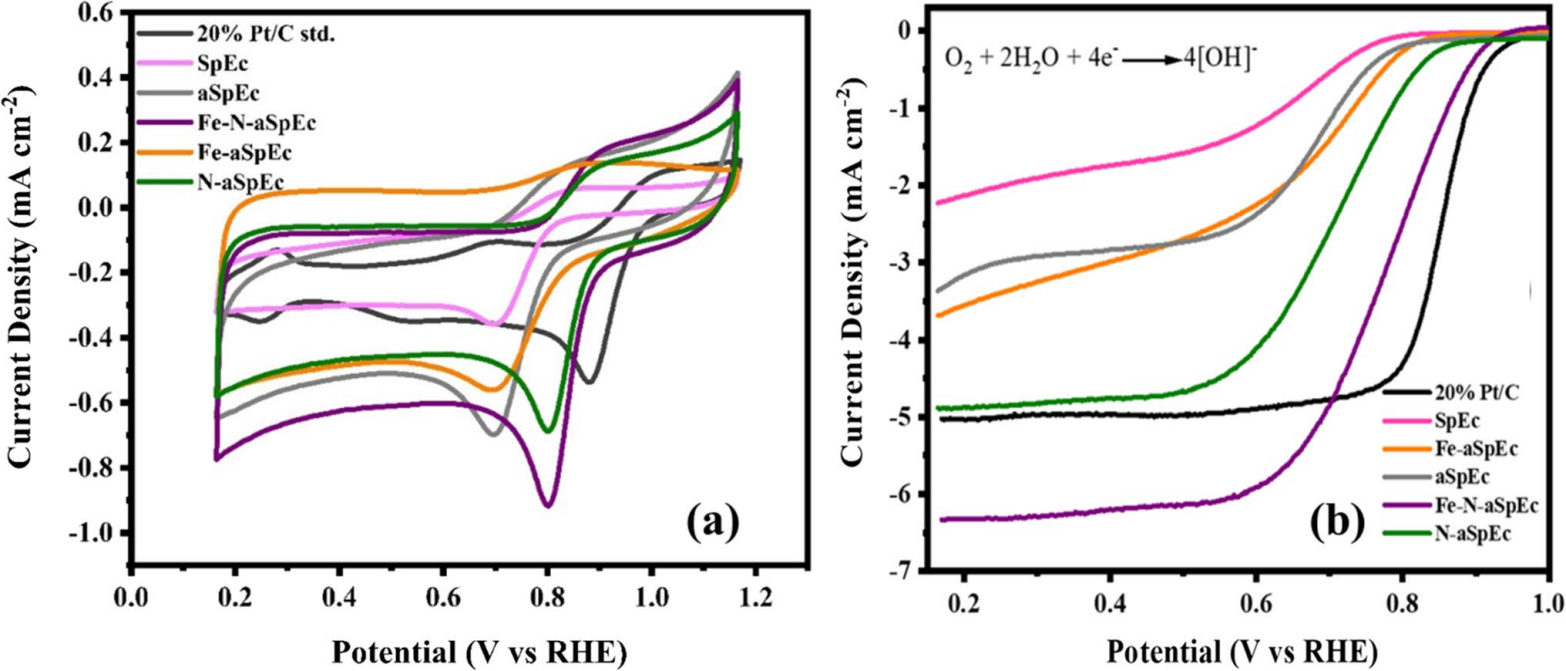
Electrochemical characterisation and comparison of 20% Pt/C standard and SpEc derived samples: (**a**) Cyclic voltammograms, (**b**) linear sweep voltammograms.

In Fig. 7a, a comparison between the ORR electrocatalytic activity of 20% Pt/C, and SpEc based electrocatalysts are shown. In this figure we compare the obtained reduction peaks of each sample containing Fe, N, and without Fe, against Pt based benchmark electrocatalyst. As expected, samples without CO_2_ activation, Fe, and N, depict an almost flat reduction peak towards high overpotentials, showing poor electrocatalytic activity for ORR. Nonetheless, with the incorporation of Fe, and N, our samples show a positive tendency towards lower overpotentials and sharper reduction peaks, implying higher charge transfer on the surface of these materials. Specifically, Fe-N-aSpEc, shows a sharper and more intense reduction peak, due to higher Fe, and N concentration according to XPS. Additionally, the high surface area of this material enhances double-layer capacitance of the material which can be measured in a working modified disk electrode without rotation. Despite the sharper and intense charge transfer shown in the CV of our most efficient sample, the reduction peak of platinum shows a peak with lower overpotential for ORR, despite the apparent lower charge transfer according to the peak magnitude of this sample, it is more important the location of this reduction peak which is correlated with lower activation energy towards ORR in alkaline media^36^.

As seen from linear sweep voltammograms (LSV) (Fig. 7b, ), Fe-N-aSpEC exhibited the best ORR catalytic properties amongst the other materials prepared. The electrocatalytic parameters obtained were found to have a marginally lower onset potential (~ 0.926 V vs. RHE) and higher diffusion limited current density (-6.3 mA cm^− 2^) compared to Pt/C standard (~ 0.972 V vs. RHE, and − 5.1 mA cm^− 2^ respectively). The material showed a half-wave potential of ~ 0.775 V vs. RHE, depicting an efficient ORR mechanism with a Tafel slope of ~ 58 mV dec^− 1^, whilst the platinum-based electrocatalyst exhibited a lower half-wave potential ~ 0.830 V vs. RHE and a characteristic Tafel slope of ~ 41 mV dec^− 1^. These findings indicate an enhancement of the ORR kinetics and mass transport of our Fe-N-aSpEC electrocatalyst compared to the benchmark material. The LSV characterisation of our best dioxygen catalysts indicates that the enhancement of the ORR activity is due to the presence of Fe coordinated with pyridinic N functionalities, graphitic N on the surface of aSpEC and the formed CNTs, as previously shown in SEM, TEM and XPS characterisations^61,65,66^. The described physical chemical characterisation of this material indicates that the surface functionalities of the Fe-N-aSpEC, specifically, the presence of Fe-N sites and pyridinic N, promote O_2_ adsorption. This reduces ORR activation energy, and graphitic N which controls mass transfer processes, thereby overall enhancing adsorption and desorption of dioxygen and hydroxyl ions during ORR. The ~ 58 mV dec^− 1^ Tafel slope (a value below 60 mV dec^− 1^) further confirms enhanced ORR kinetics, in particular, suggesting a preferential 4 e- pathway for the Fe-N-SpEC biomass supported catalysts. Additionally, electrochemical impedance spectroscopy was measured on 20% Pt/C and Fe-N aSpEc materials. As expected, Pt standard showed a lower charge transfer resistivity of ~ 40 ohms, whilst our electrocatalysts depicted a resistivity of ~ 60 ohms, this in accordance with Tafel slope analysis, implies that ORR kinetics are faster in Pt based standard rather than in our most efficient sample (Fe-N-aSpEC) (see Figure S5)^67–70^.

**Fig. 8:**
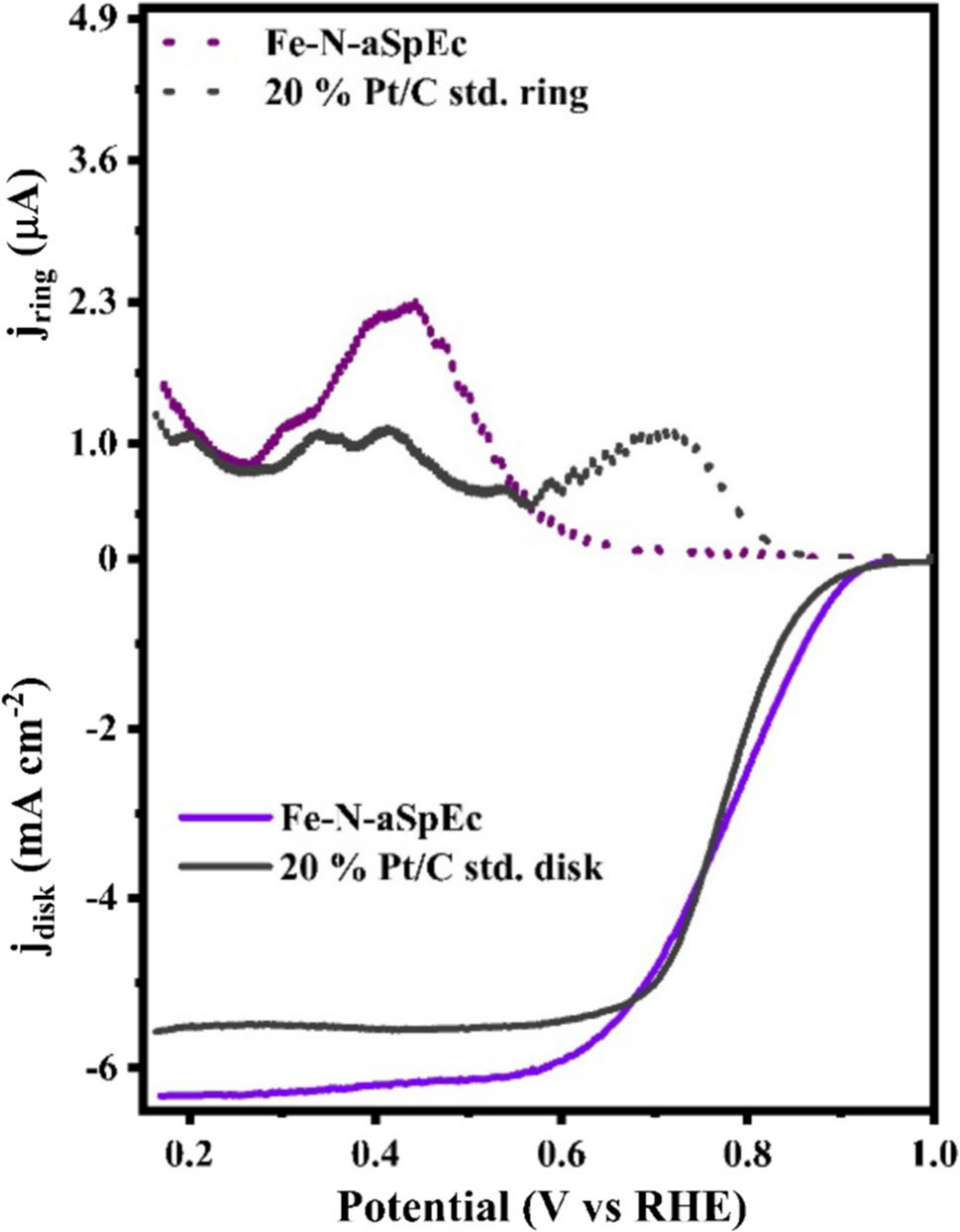
RRDE linear sweep voltammograms comparison of 20% Pt/C and Fe-N-aSpEc. Note that ring current appears noisier than disk current. This is attributed to mass transport and disk rotation speed which can induce an abrupt adsorption of Hydrogen peroxide and desorption of [OH]^-^ ions from the surface of this secondary electrode which generate noise in the µA range.

Additional to the ORR electrochemical characterisation via RDE technique, we used rotating ring disc electrode (RRDE) technique (Fig. 8) to get more information about the electrochemical process happening on the surface of 20% Pt/C standard and SpEC based electrocatalysts. For the platinum benchmark electrocatalyst and the aSpEC based electrocatalysts, the ring current indicate the formation of [OOH]- at potentials bellow ~ 0.70 V vs. RHE. This being a typical behaviour of poly-crystalline Pt nanoparticles^71,72^. In contrast, the Fe-N-aSpEC electrocatalyst showed an increment on the ring current at ~ 0.76 V vs. RHE, in the region of mass transport overpotential, suggesting the formation of peroxide species just within that region of electrochemical potentials. This behaviour indicates an effective adsorption of bulk O_2_ at the catalytic sites of the material, and a subsequential formation of [OH]- preferentially at electrochemical potentials towards the thermodynamically predicted potential for ORR (~ 1.23 V vs. RHE).

**Fig. 9:**
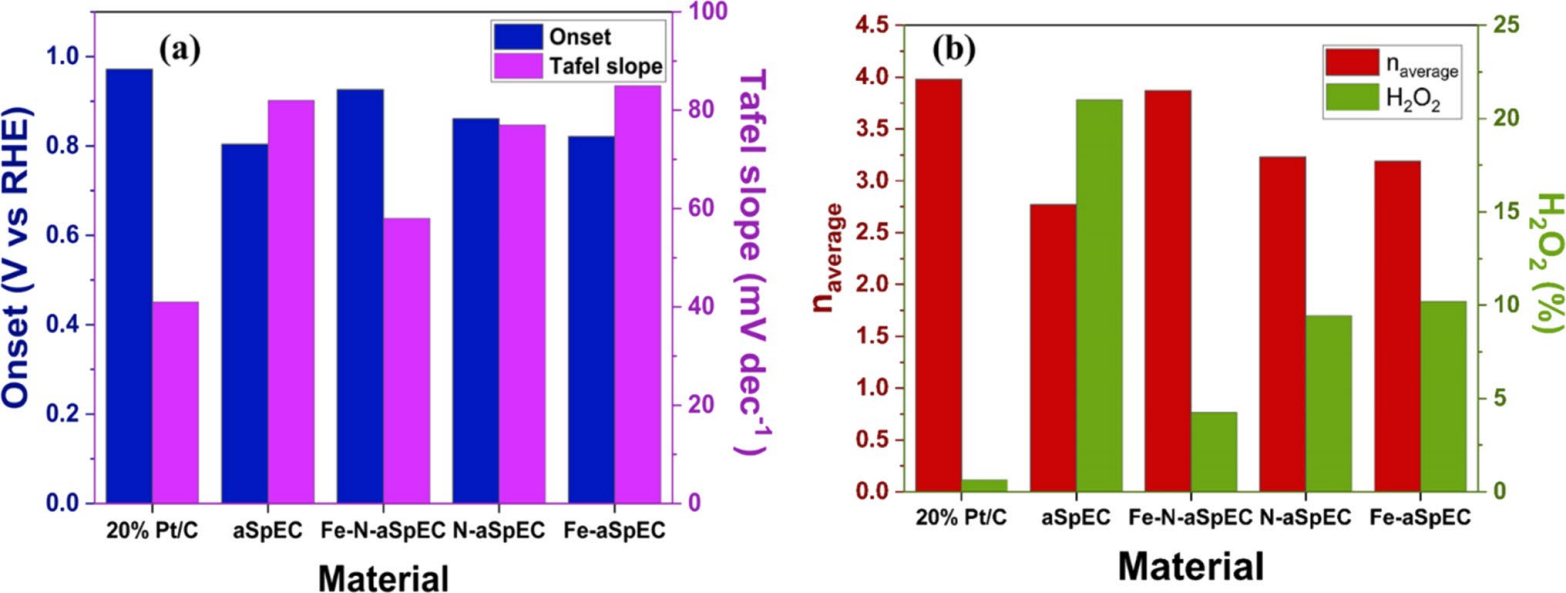
Comparison of the key electrocatalytic properties of the different samples, (**a**) bar chart comparing the onset potential and Tafel slope, and (**b**) bar chart comparing effective electron transfer number (n) and hydrogen peroxide yield.

Figure 9a presents a summary comparing the onset potential and Tafel slope of all samples, while Fig. 9b provides a comparison of the effective electron transfer number (n) and hydrogen peroxide yield for the same samples. At potentials bellow ~ 0.76 V vs. RHE, the RRDE calculations indicate a mixed 4 e- and 2 e- pathways, preferentially the former pathway. This is further supported by the determined effective electron transfer number (n) value of 3.87, and low hydrogen peroxide yield of 4.25% (Fig. 9b). Furthermore, efficient O_2_ electrocatalysis at low overpotentials can be reasonably attributed to the presence in high concentration of Fe-N, pyridinic and graphitic N on the surface of the material according to XPS analysis. These active sites are well known to decrease activation energy, enhance kinetics of reaction (pyridinic N), and promote dioxygen adsorption and desorption (graphitic N). The 2 e- pathway can be reasonably attributed to the presence of acridine and carbazole N active sites that promote this specific pathway due to the electron conformation of these nitrogen moieties. Hence, the increment on ring current indicates a deviation of 2e^−^ pathway at high overpotentials, impacting on a decrease of the effective electron transfer number (n)^61,65–67^. The mathematical models used to calculate these parameters have also been added to the Supplementary material (Table S1).

**Fig. 10:**
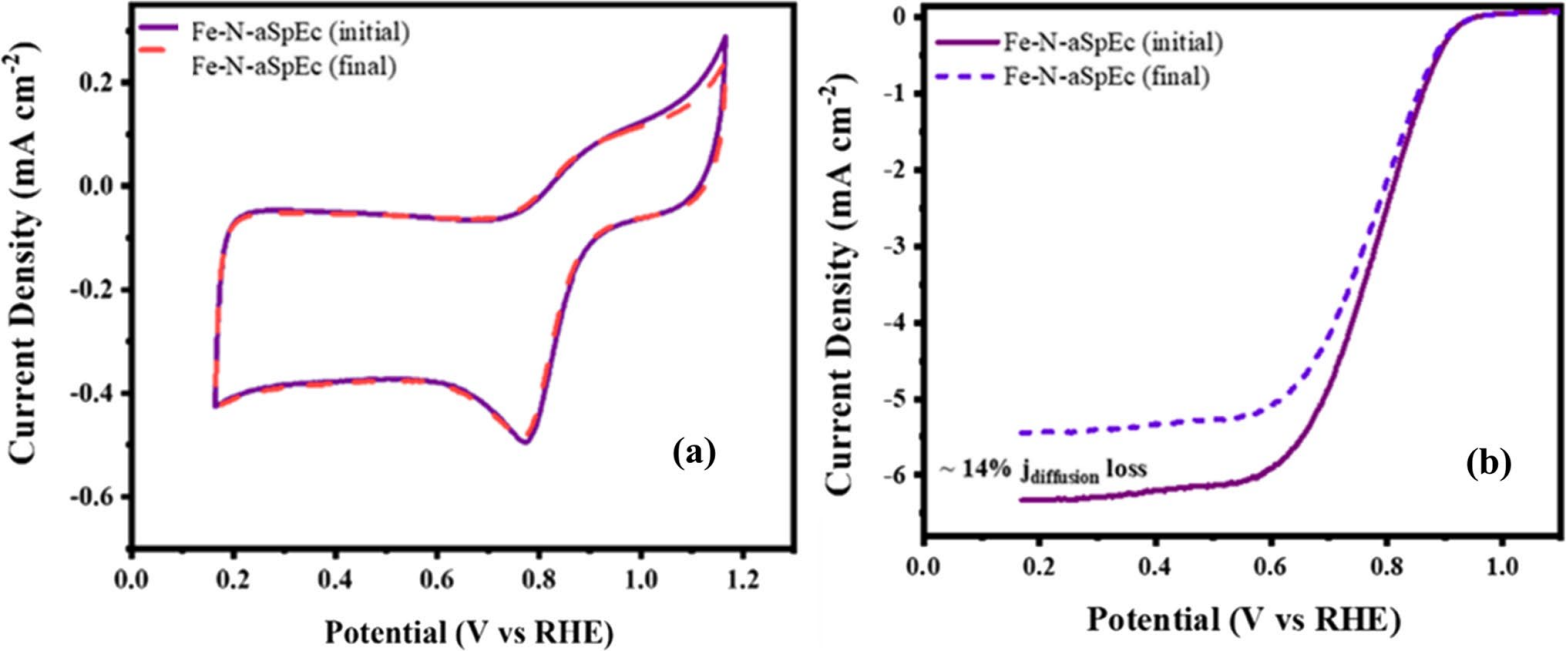
Stability testing of Fe-N-aSpEc was measured by cycling the material 3,000 times under alkaline conditions, (**a**) initial and final CVs of Fe-N-aSpEc after stability testing, and (**b**) initial and final LSVs of Fe-N-aSpEc after accelerated degradation testing.

The stability testing of Fe-N-aSpEc was measured by cycling the material 3,000 times under alkaline conditions between the reductive potentials of 0.95 and 0.45 V vs. RHE^73,74^. In Fig. 10a and b, the stability CV and LSV results of Fe-N-aSpEC ORR electrocatalysts are shown. For both materials, the initial LSV polarisation curves show the characteristic ORR activity that was previously determined. After 3,000 cycles, the Fe-N-aSpEC showed a neglectable difference between the initial and final CV measurements, demonstrating a remarkable stability, which can be reasonably attributed to the robust structure of the SpEC and the N-doped CNTs. The half-wave potential shift on the last LSV measured was relatively contained. Nonetheless, a 14% loss on the diffusion limited current density, governed by mass transfer process, indicates that owing to peroxide formation, our material exhibits different adsorption and desorption rates of OH- and H_2_O_2_, as well as a peroxide poisoning of graphitic N active sites that have been reported as the main factors in mass transport controlled processes^60,75,76^. Overall, the general preservation of onset and half-wave potentials implies that our material remains very active towards ORR, while the Fe-N, and pyridinic N active sites are preserved despite the accelerated aging process induced by electrochemical cycling. This also suggests that Fe-N-aSpEC might be suitable to fabricate a gas diffusion layer (GDL) supported cathode for alkaline fuel cells and primary metal air batteries^61,77,78^. A comparison of physical-chemical properties of recently reported electrocatalysts comparable to our Fe-N-aSpEC is shown in Table S2.

## Conclusions

We employed a simple, sustainable, and efficient method for the synthesis of iron-nitrogen co-doped 3D porous carbon electrocatalyst for the ORR by utilising readily available and bio-sourced SpECs as catalyst support and carbon source, and melamine and iron acetate as the source of nitrogen and iron respectively. Combination of absorbed melamine on to the surface of the iron particles and diffused carbon in the form of liquid metastable carbides at high temperatures, was able to form highly desirable nitrogen-doped CNT onto the surface of aSpEC. Our electrocatalyst depicted an efficient activity and stability towards ORR in alkaline conditions, with comparable onset (just ~ 46 mV offset from Pt) and half-wave potentials to Pt, as well as an outstanding diffusion limited current density. The efficient ORR performance can be credited to the extensive N doping of the graphitic structures present on the surface of the SpEC and an open ordered structure, which allow for exposed active sites and for a fluent conductive environment for electron transfer, with vast networks for electrolyte and O_2_ diffusion. Additionally, RRDE characterisation supports the previous findings by demonstrating an effective electron transfer number of 3.87 and a low peroxide yield of 4.25%, signifying that the synthesis process of the material was successfully optimised to achieve an ideal concentration of Fe and N active sites, as well as large surface area provided by the activation processes. Finally, we assessed the stability of our material to provide indications on the suitability for larger scale industrial applications, to demonstrate that the robust nature of SpEC and N-doped CNTs, combined with a high surface area, preserve active sites which facilitates ORR kinetics (Fe-N and pyridinic N). The high degree of graphitisation of core-shell and structures encapsulating the active metal nanoparticles were key to improving the stability of the catalyst by preventing metal agglomeration and metal leaching. Our work shows a sustainable and economical efficient option for manufacturing ORR cathodes that can be used in alkaline fuel cells, and metal air batteries. Overall, the presented method demonstrates a promising potential for the conversion of abundant and cost-effective biomass wastes into highly desirable, effective catalysts.

## Electronic Supplementary Material

Below is the link to the electronic supplementary material.

Supplementary Material 1

## Data Availability

The datasets used and/or analysed during the current study are available from the corresponding author on reasonable request.

## Declarations

### Policy compliance

This study does not include species endangered or at risk of extinction, and was conducted in accordance with relevant institutional, national, and international guidelines in particular the IUCN Policy Statement on Research Involving Species at Risk of Extinction and the Convention on the Trade in Endangered Species of Wild Fauna and Flora.
