# Supplementary material for: Fe-N catalyst derived from and supported on Lycopodium clavatum sporopollenin exine capsules for the oxygen reduction reaction
Source: Sci Rep. 2024 Oct 30;14:26052. doi: 10.1038/s41598-024-77780-1 (PMC11522689; doi:10.1038/s41598-024-77780-1)
Supplement: Supplementary file 1 — Supplementary Material 1 [file 41598_2024_77780_MOESM1_ESM.docx]

**Supplementary Material**

**Fe-N Catalyst Derived from and Supported on *Lycopodium Clavatum* Sporopollenin Exine Capsules for the Oxygen Reduction Reaction**

Waqas Malik^a^, Jorge Pavel Victoria Tafoya^a^, Szymon Doszczeczko^a^, Ana Belen Jorge Sobrido^a^, Andrew N. Boa^b^, Roberto Volpe ^a^*

^a^School of Engineering and Materials Science, Queen Mary University of London, London E1 4NS, UK

^b^Department of Chemistry, University of Hull, Hull, HU6 7RX, UK

*Corresponding Author: Dr Roberto Volpe, [r.volpe@qmul.ac.uk](mailto:r.volpe@qmul.ac.uk)


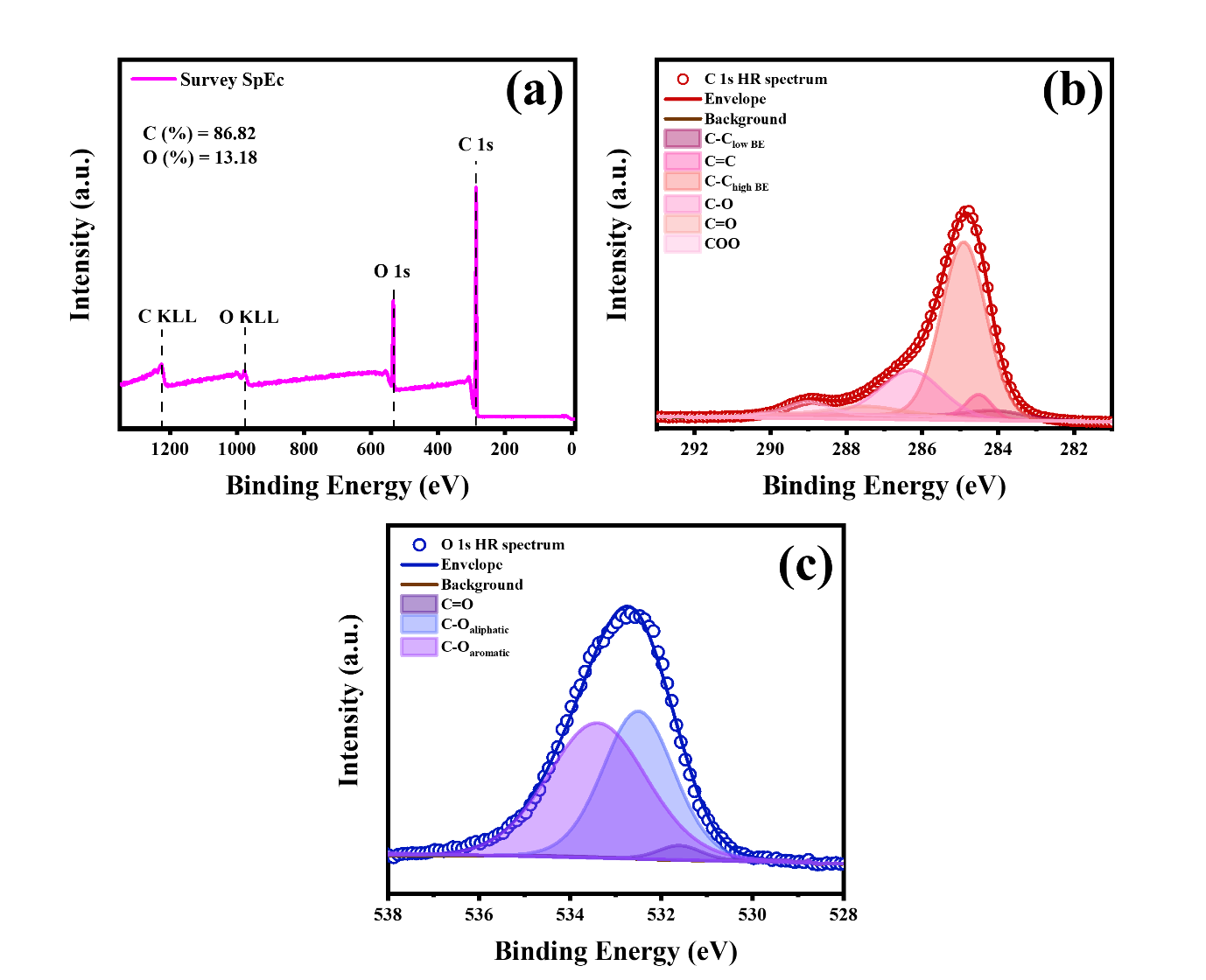


**Figure S1.** X-ray photoelectron spectroscopy characterisation of SpEC sample: (a) survey spectrum, (b) C 1s spectrum, and (c) O 1s spectrum.


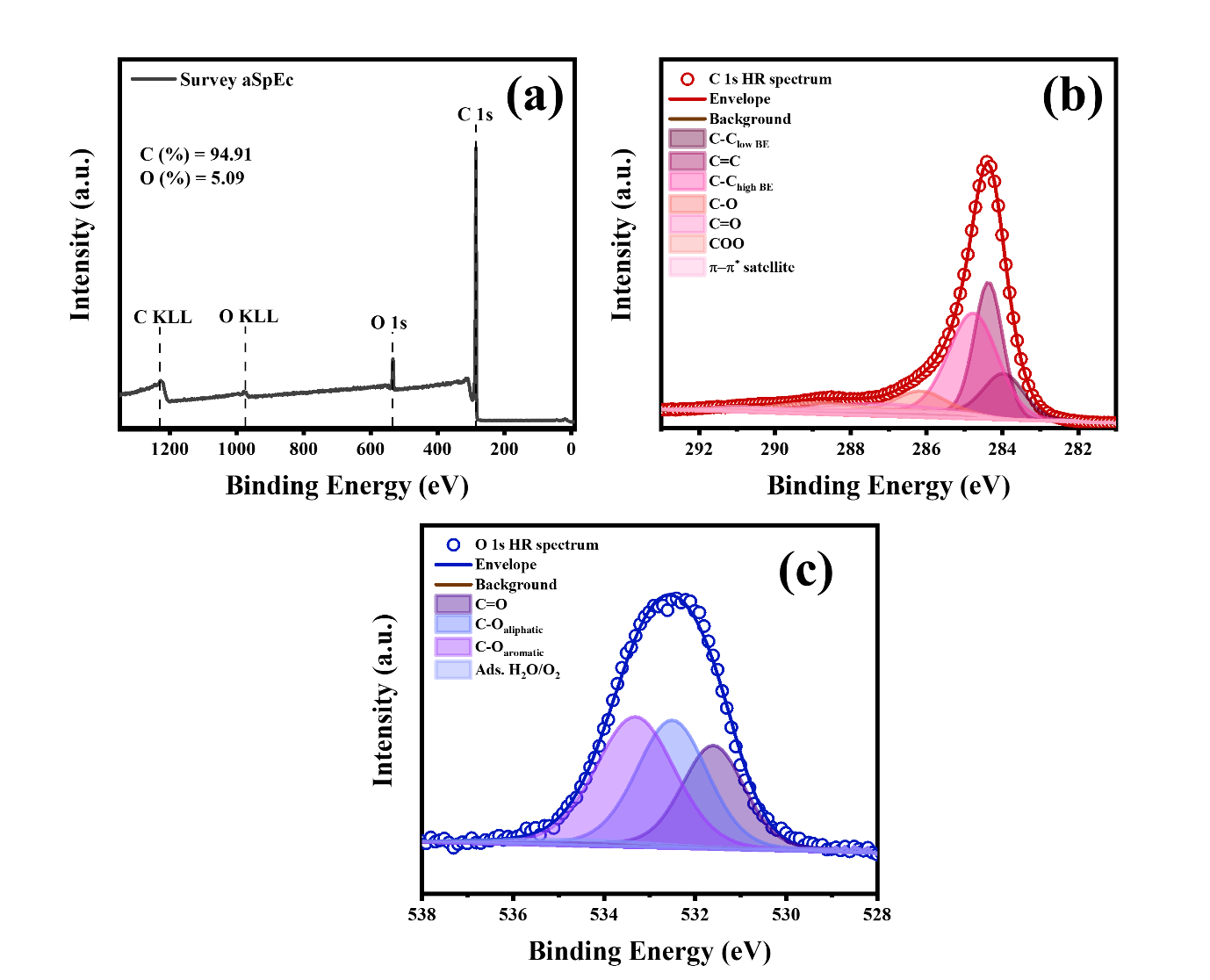


**Figure S2**. X-ray photoelectron spectroscopy characterisation of aSpEC sample: (a) survey spectrum, (b) C 1s spectrum, (c) O 1s spectrum.


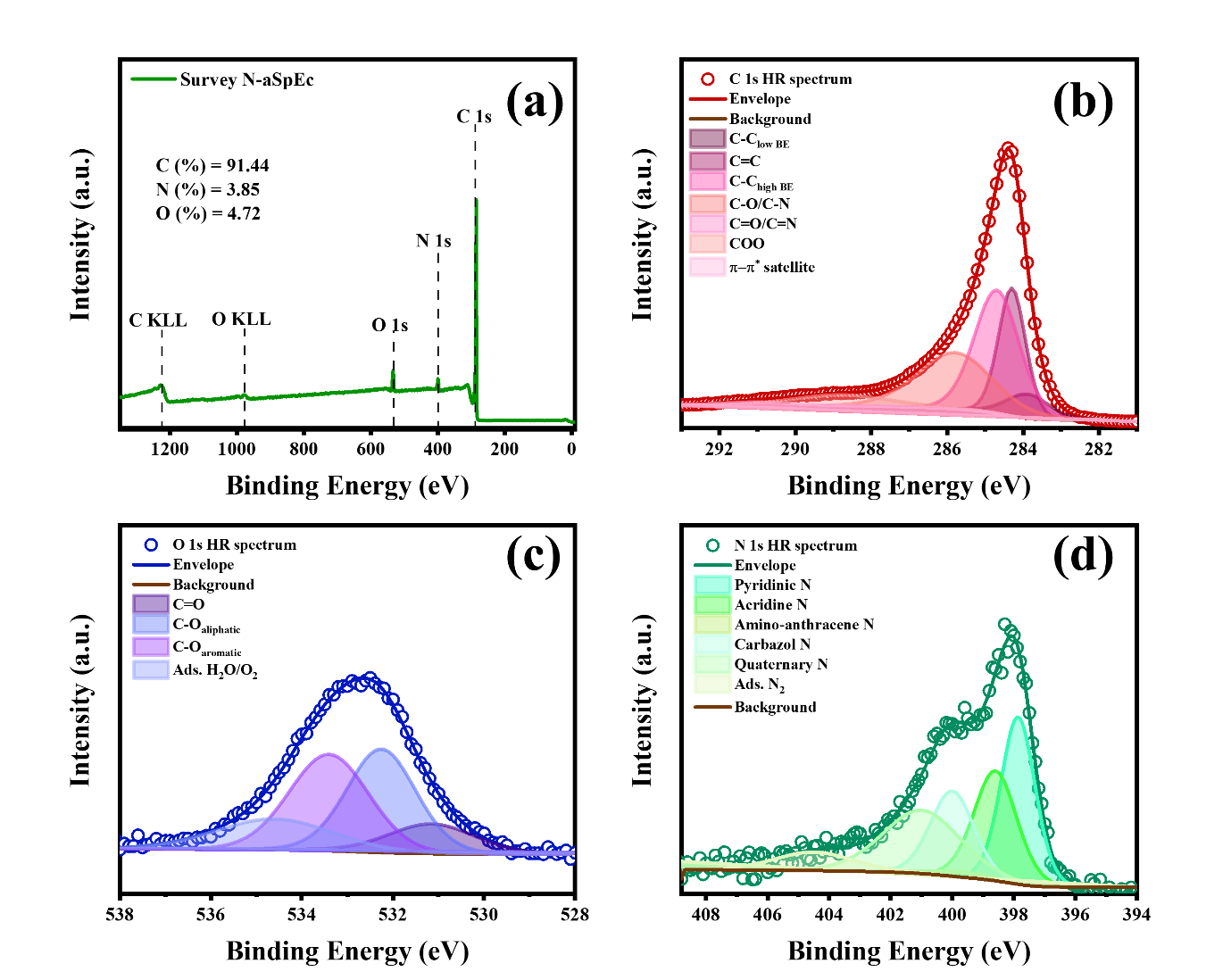


**Figure S3**. X-ray photoelectron spectroscopy characterisation of N-aSpEC sample: (a) survey spectrum, (b) C 1s spectrum, (c) O 1s spectrum, and (d) N 1s spectrum.


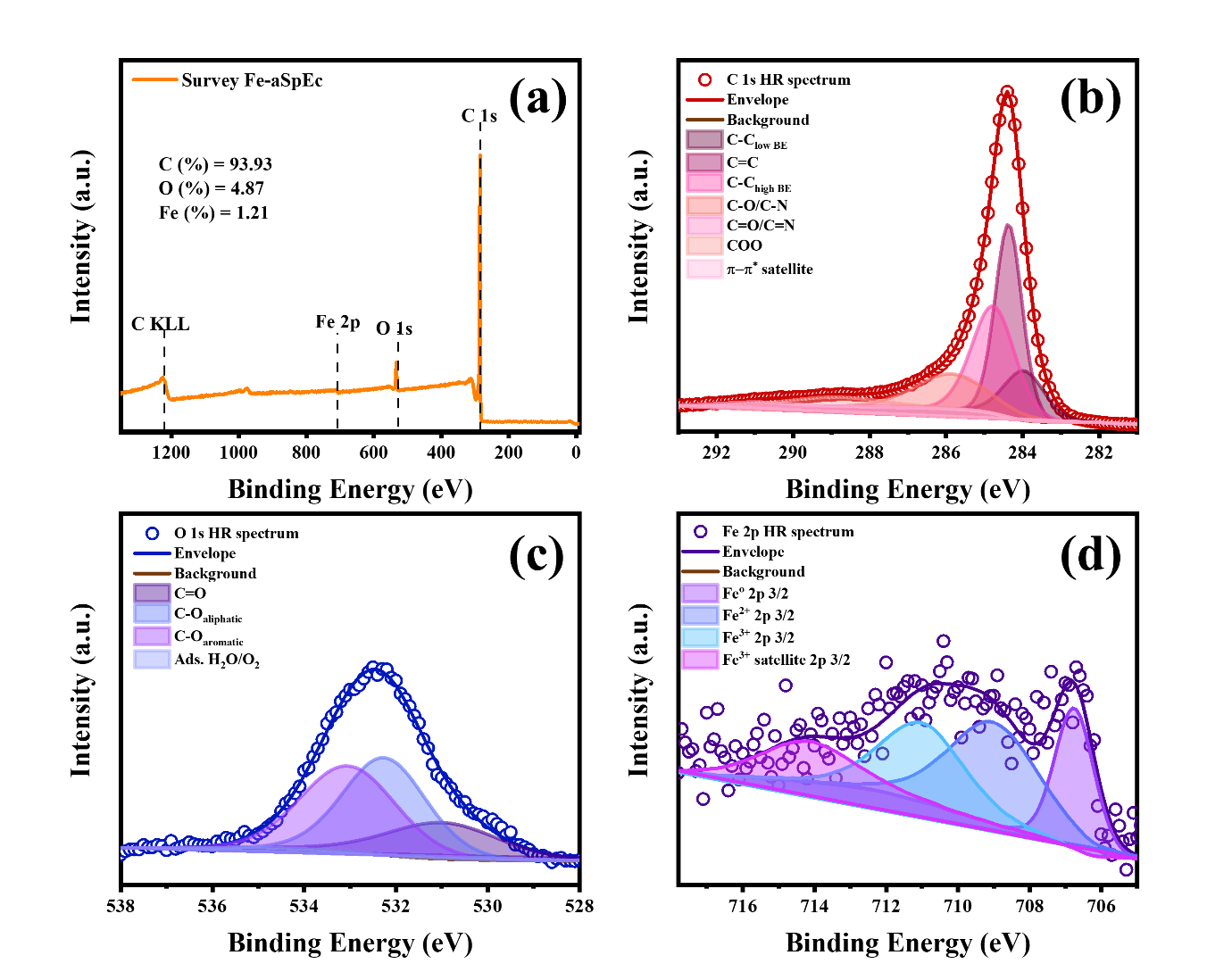


**Figure S4.** X-ray photoelectron spectroscopy characterisation of Fe-aSpEC sample: (a) survey spectrum, (b) C 1s spectrum, (c) O 1s spectrum, and (d) Fe 2p spectrum.


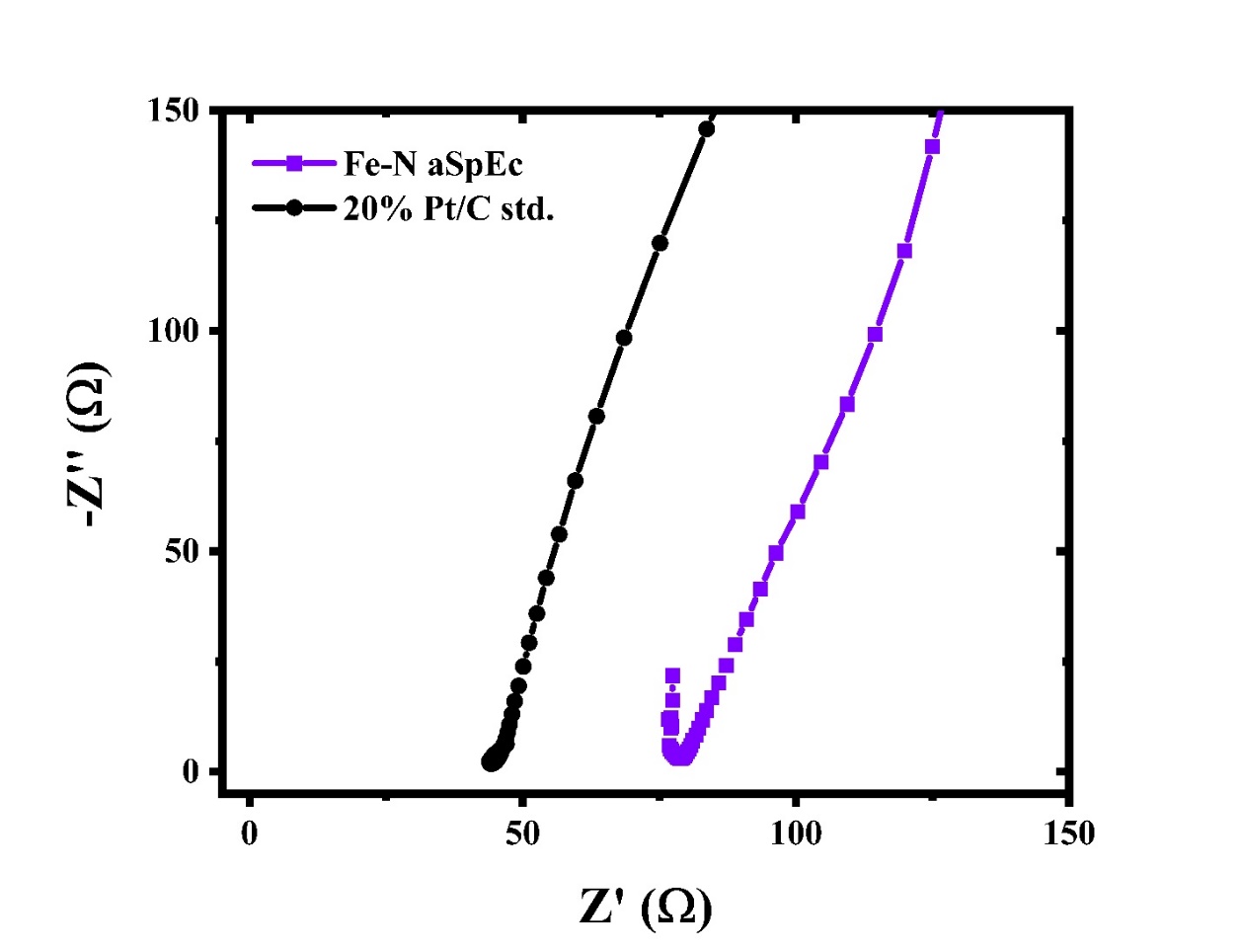


**Figure S5**. Showing the impendence spectroscopy of the synthesised Fe-N aSpEc electrocatalyst and the Pt/C standard.


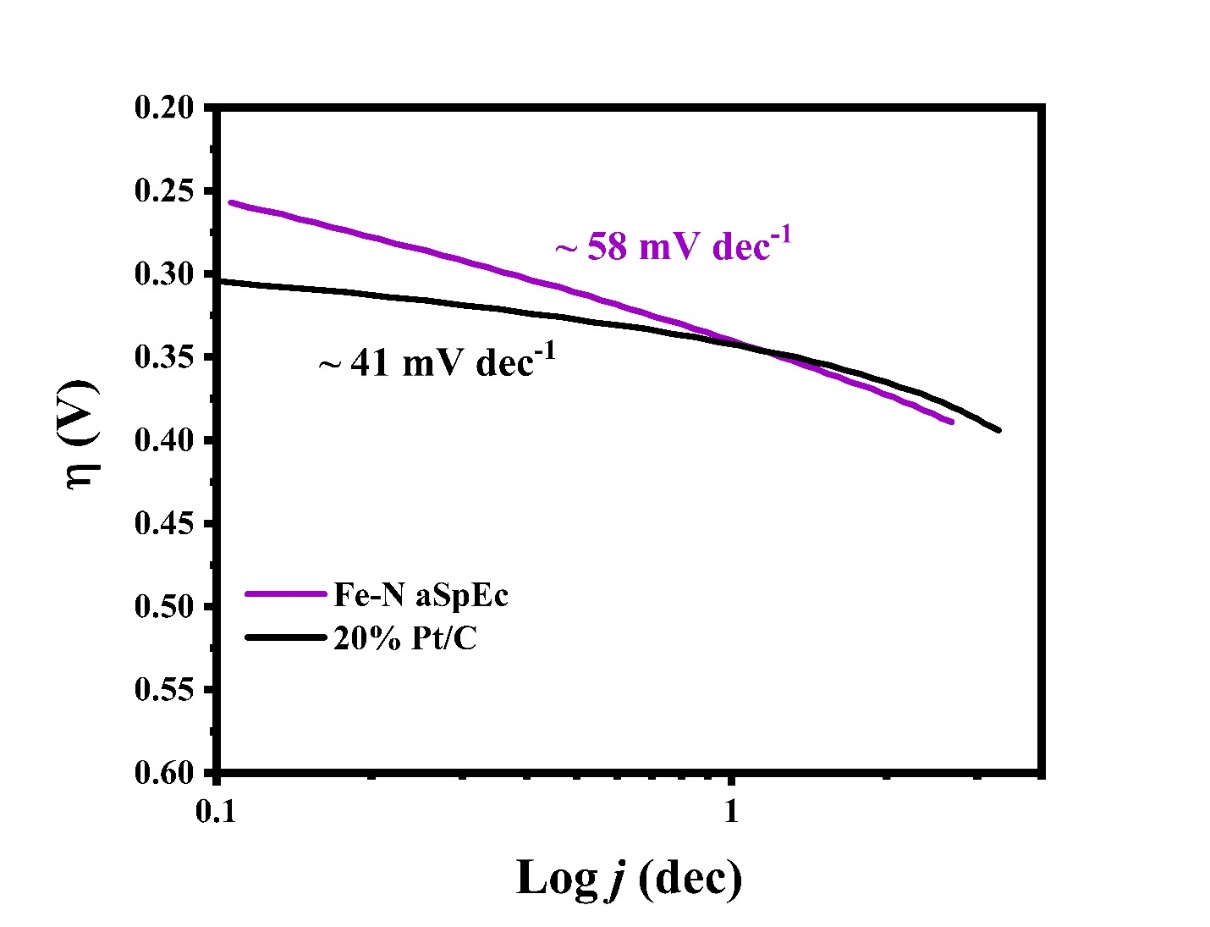


**Figure S6**. Showing Tafel slope of the synthesised Fe-N aSpEc electrocatalyst and the Pt/C standard.

**Table S1.** Equations used to determine effective electron transfer number (n) and hydrogen peroxide yield

| $n=\frac{4\cdot I_{D}}{I_{D}+\frac{I_{R}}{N}}$ | *Equation 1* |
| --- | --- |
| $H_{2}O_{2}\left( \% \right)=\frac{2\cdot\frac{I_{R}}{N}}{I_{D}+\frac{I_{R}}{N}}\cdot100$ | *Equation 2* |

Note: I_D_ is the current measured in amperes at the disk (glassy carbon electrode modified with our catalyst), I_R_ is the current measured in amperes at the ring (attributed to H_2_O_2_ formation), and N is the collection efficiency of the ring secondary working electrode, which in our case was 23% according to the manufacturer (Metrohm®).

**Table S2**. Comparison of physical-chemical properties of recently reported electrocatalysts

| Material | Synthesis | Surface Area | Dopant | Atomic % | *E_onset_*  (V vs RHE) | *E_1/2_*  (V vs RHE) | *n* | Ref. |
| --- | --- | --- | --- | --- | --- | --- | --- | --- |
| Fe-N-aSpEC | Thermal activation of biomass/  impregnation/  pyrolysis | 496 | Fe  N | 0.71  2.29 | 0.926 | 0.775 | 3.87  average | This work |
| Fe, N- doped nanosheets | Biomass soot production/ impregnation/ pyrolysis | 374 | Fe  N | 1.28  4.79 | 0.990 | 0.850 | 3.72  average | ^1^ |
| Fe-N co-doped porous carbon | One step pyrolysis of biomass and ferric nitrate | 520.9 | Fe N | 1.79  6.24 | 0.989 | 0.854 | 3.65  Average | ^2^ |
| Single atom Fe coordinated, N doped hierarchically porous C | Confinement effect of porous carbons | 1,449.1 | Fe  N | 0.48  3.27 | NA | 0.870 | ~ 4 | ^3^ |
| Fe-N co-doped nanospheres | Hydrothermal carbonisation of biomass/ pyrolysis | 70 | Fe  N | 1.88 5.77 | 0.940 | 0.790 | 3.7 ≤ n ≤ 3.95  @  0.2 ≤ϕ ≤1 V vs RHE | ^4^ |

1. Luo, X. *et al.* Biomass derived Fe,N-doped carbon material as bifunctional electrocatalysts for rechargeable Zn-air batteries. *Journal of Alloys and Compounds* 888, 161464 (2021).

2. Liu, Y. *et al.* Soybean straw biomass-derived Fe–N co-doped porous carbon as an efficient electrocatalyst for oxygen reduction in both alkaline and acidic media. *RSC Advances* 10, 6763–6771 (2020).

3. Zhang, Z., Gao, X., Dou, M., Ji, J. & Wang, F. Biomass Derived N-Doped Porous Carbon Supported Single Fe Atoms as Superior Electrocatalysts for Oxygen Reduction. *Small* 13, 1604290 (2017).

4. Feng, J. *et al.* Iron, Nitrogen Co-Doped Carbon Spheres as Low Cost, Scalable Electrocatalysts for the Oxygen Reduction Reaction. *Adv Funct Mater* 31, 2102974 (2021).
